# Ruptured hepatic hydatid cyst causing cholangitis

**DOI:** 10.1590/0037-8682-0530-2022

**Published:** 2022-12-16

**Authors:** Ramazan Orkun Önder, Tumay Bekci, Demet İlkim Yayla

**Affiliations:** 1 Giresun University, Faculty of Medicine, Department of Radiology, Giresun, Turkey.

**FIGURE 1:**
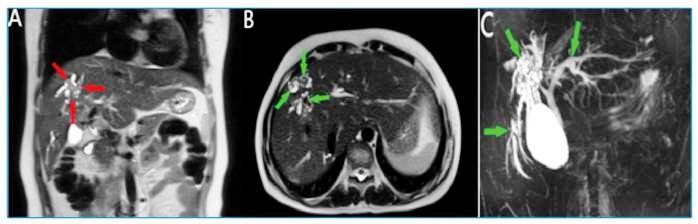
**(A)** Heterogeneously hyperintense lesion area in the liver dome observed on MRCP (red arrows). **(B)** Dilatation of the intrahepatic bile ducts and appearances of the hypointense content in their lumens (green arrows). **(C)** Dilated bile ducts (green arrows).

A 37-year-old woman was admitted to the emergency department because of fever and abdominal pain. The medical history showed a diagnosis of a liver hydatid cyst (HC) 1 year before. Physical examination showed a body temperature of 38.3°C. Laboratory tests revealed elevated C-reactive protein (55 mg/L), direct bilirubin (4.53 mg/dL), and total bilirubin (5.24 mg/dL) levels. Abdominal ultrasonography revealed an irregularly contoured, heterogeneously hypoechoic lesion in the anterior segment of the right liver lobe and dilatation in the intrahepatic bile ducts. Therefore, magnetic resonance cholangiopancreatography (MRCP) was performed. It showed a heterogeneously hyperintense lesion in the liver dome. In addition, dilatation was detected in the intrahepatic bile ducts at the level of the right and left liver lobes, with hypointense content in their lumens (Figure 1). Based on the findings, spread of primary liver hydatid disease to the intrahepatic bile ducts and cholangitis complications were suspected. The patient was treated with endoscopic retrograde cholangiopancreatography, and the membranes observed in the bile ducts were cleaned. Subsequently, the patient was discharged and started on albendazole. In approximately 5%-30% of patients with an untreated HC, the intracystic pressure exceeds the pressure in the biliary tract, causing the HC to rupture or fistulize the bile ducts, spontaneous decompression of the cysts, and cholangitis^1^ ^-^ ^3^. The complaints of fever and pain in the upper right abdominal quadrant in a known case of HC may indicate a rupture in the biliary tract.
